# Social cognitive determinants of exercise behavior in the context of behavior modeling: a mixed method approach

**DOI:** 10.1177/2055207618811555

**Published:** 2018-11-14

**Authors:** Kiemute Oyibo, Ifeoma Adaji, Julita Vassileva

**Affiliations:** University of Saskatchewan, Saskatoon, Canada

**Keywords:** Social cognitive model, bodyweight exercise, behavior modeling, fitness app, persuasive technology, gender difference

## Abstract

Research has shown that persuasive technologies aimed at behavior change will be more effective if behavioral determinants are targeted. However, research on the determinants of bodyweight exercise performance in the context of behavior modeling in fitness apps is scarce. To bridge this gap, we conducted an empirical study among 659 participants resident in North America using social cognitive theory as a framework to uncover the determinants of the performance of bodyweight exercise behavior. To contextualize our study, we modeled, in a hypothetical context, two popular bodyweight exercise behaviors – push ups and squats – featured in most fitness apps on the market using a virtual coach (aka behavior model). Our social cognitive model shows that users’ perceived self-efficacy (β_T_ = 0.23, *p* < 0.001) and perceived social support (β_T_ = 0.23, *p* < 0.001) are the strongest determinants of bodyweight exercise behavior, followed by outcome expectation (β_T_ = 0.11, *p* < 0.05). However, users’ perceived self-regulation (β_T_ = –0.07, *p* = n.s.) turns out to be a non-determinant of bodyweight exercise behavior. Comparatively, our model shows that perceived self-efficacy has a stronger direct effect on exercise behavior for men (β = 0.31, *p* < 0.001) than for women (β = 0.10, *p* = n.s.). In contrast, perceived social support has a stronger direct effect on exercise behavior for women (β = 0.15, *p* < 0.05) than for men (β = −0.01, *p* = n.s.). Based on these findings and qualitative analysis of participants’ comments, we provide a set of guidelines for the design of persuasive technologies for promoting regular exercise behavior.

## Introduction

Recent studies of the most popular topics in the health and fitness domain show that bodyweight exercises have gained traction and attention among health and fitness enthusiasts worldwide. For example, in a global survey of the trending health and fitness topics, bodyweight exercise has consistently featured in the top four positions of the fitness trend chart in the last 3 years.^1–3^ Bodyweight exercise is the use of one’s body weight as resistance as opposed to free weights or exercise equipment during a workout. There are a number of reasons for its popularity worldwide. First, it is inexpensive in the sense that it does not require the owning of equipment or signing up to become a member of a gym. Second, it can be performed in the comfort of one’s home (e.g. bedroom, sitting room, etc.). Third, it can be done when one is away from home (e.g. in a hotel room). Finally, bodyweight exercise offers a number of health benefits, which include gaining of strength, building of muscles, improvement of cardiovascular fitness, and burning of fat.^4,5^ Overall, bodyweight exercise is an effective way to improve balance, stability and flexibility.^6^ Hence, it has become important for researchers to investigate its determinants with the specific aim of designing persuasive applications to support its performance anywhere and anytime.

In general, physical inactivity has been pin-pointed as one of the main causes of non-communicable diseases, such as strokes, hypertension, type 2 diabetes, etc., which account for 6% of global mortality annually.^7^ Research has shown that most adults (within the age group of 18–64 years) worldwide do not meet the minimum recommendation of at least 150 minutes of moderate-intensity aerobic physical activity per week, or its equivalent of at least 75 minutes of vigorous-intensity aerobic physical activity per week.^7,8^ Moreover, research has shown that, although most people are aware of the importance and benefits of physical activity, they lack the willpower or motivation to exercise regularly.^9^ Apart from the lack of motivation, lack of time due to other priorities^10^ and lack of access to recreational facilities, e.g. gym,^11^ are among the main reasons why people do not exercise regularly. Thus, there is a need for health practitioners, researchers and persuasive technology (PT) designers to promote physical activity in the context of an individual’s circumstances and environment.^11^ We argue that encouraging home-based bodyweight exercise might be one way to tackle the challenges of lack of time and access to physical activity facilities. The reason for this is that home-based bodyweight exercise does not require people to leave their home; neither does it require them to possess exercise equipment, which may be unaffordable to some people and could be a barrier to physical activity.^11^ Research has shown that PTs can be used as an effective support system to motivate and facilitate positive behavior change in humans,^12^ especially those who are willing and open to change.^13^

Thus, to assist PT researchers and designers in developing well-informed behavior change support systems in this area, we conducted an empirical study of the social cognitive theory (SCT) determinants of bodyweight exercise behavior in the context of behavior modeling in a fitness app. In recent years, behavior modeling has been found to be one of the most commonly used behavior change techniques in most fitness apps in the marketplace.^14^ By definition, behavior modeling entails the demonstration of the correct performance of a given behavior by an expert to an observer.^15–17^ It is a form of vicarious modeling, which could be carried out in a real-life environment, such as a classroom, by a real person, or in a simulated (virtual) environment, such as a video, by a virtual coach or role model. On the other hand, SCT is one of the most widely applied behavioral theories for promoting health interventions.^18^ The link between behavior modeling and SCT is ‘observational learning’. In particular, observational learning is at the core of the social learning theory (SLT), which later developed into the SCT. It posits that through behavior modeling, people are able to observe the performance of a given behavior and reproduce it subsequently. More specifically, it holds that ‘if individuals see successful demonstration of a behavior, they can also complete the behavior successfully’.^18^ This is made possible through cognitive processes which motivate and/or mediate human behaviors.^19^ However, in the context of behavior modeling in the fitness domain, there is limited research on how the core SCT factors, which are impacted by the perceived persuasiveness of behavior models,^20^ in turn, influence exercise behavior performance.

To bridge this gap and advance the current research in this area of PT, we modeled the SCT determinants of bodyweight exercise behavior, using videos of behavior models performing push-ups and squats as a case study. Our study was based on a sample of 659 participants resident in North America. The results of our structural equation modeling (SEM) show that the observers’ perceived self-efficacy (β_T_ = 0.23, *p* < 0.001) and perceived social support (β_T_ = 0.23, *p* < 0.001) are the strongest determinants of bodyweight exercise behavior performance, followed by outcome expectation (β_T_ = 0.11, *p* < 0.05). Perceived self-regulation (β_T_ =−0.07, *p* = n.s.) turns out not to be a determinant of bodyweight exercise behavior performance. Comparatively, our SCT model shows that, for men, perceived self-efficacy is a stronger determinant (motivator) than perceived social support, while, for women, perceived social support is a stronger determinant than perceived self-efficacy. Finally, based on these findings and qualitative analysis of participants’ comments, we provide a set of design guidelines to help fitness app designers to develop more effective PT interventions in the fitness domain.

## Background

In this section, we provide an overview of SCT and observation learning. For brevity, in the rest of the paper, for the most part, we will omit the qualifier ‘perceived’ from the names of the SCT factors.

### Social cognitive theory

SCT is a behavior theory of human motivation and action. It is an offshoot of the SLT^21^ proposed by Bandura^22^ to explain the various internal and external processes (cognitive, vicarious, self-reflective and self-regulatory) that come into play in human psychosocial functioning. It is organized within a causation framework known as the triadic reciprocal determinism, which states that cognitive, behavioral and environmental factors dynamically interact with one another in a reciprocal fashion to shape human behavior.^23^ For example, with respect to exercise behavior, self-efficacy, self-regulation and outcome expectation are typical examples of cognitive factors which shape behavior, while social support is an example of environmental factors. Self-efficacy refers to the belief in one’s ability to perform a given behavior. It is regarded as the strongest (proximal) determinant of behavior change.^24^ Self-regulation is the control and management of one’s behavior through planning, setting goals and self-monitoring of one’s performance. Outcome expectation is the belief one holds about the consequences of a behavior, which could be positive or negative. Finally, social support refers to the assistance people get from others towards the performance of a behavior. For the purpose of our paper, these theoretical determinants of behavior are examined at the level of perception in the context of behavior modeling aimed at motivating exercise behavior change.

### Observational learning

**Figure 1.**
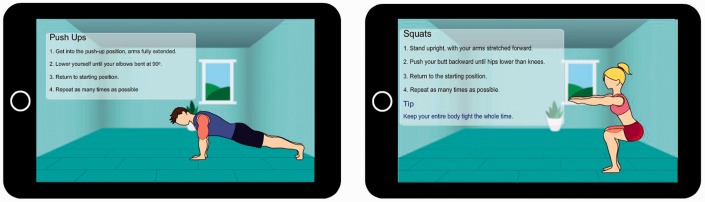
Videos of behavior models demonstrating push-up and squat exercises.^20^

Observational learning refers to the acquisition of knowledge through observation. According to Bandura,^22^ ‘observational learning enables humans to develop their knowledge and skills through information conveyed by modeling influences’ (p. 25). SCT holds that much of human knowledge is acquired through observational learning. In particular, it states that people intentionally or unintentionally learn by observing the behaviors of others (models) and their consequences. Moreover, it holds that people may choose to replicate a behavior depending on whether they are rewarded or punished for it. Electronic technologies (e.g. television, radio, etc.) are examples of mass media through which behavior models can transmit new ways of thinking and behaving to a critical mass of people in the society at large with the aim of changing attitudes and behaviors.^22^ In more recent times, social media and gamified PTs have become popular media, also known as socially influencing systems,^25^ aimed at motivating behavior change, including engagement in targeted behaviors such as cycling,^26^ healthy eating,^27,28^ physical activity,^20^ etc. In the context of our study, our simulated behavior models (in a prototyped fitness application), shown in Figure 1, represent virtual social agents of change,^29^ with the observers of the modeled exercise behavior being the targeted audience.

## Related work

A number of studies have been carried out with respect to the social cognitive model of behavior, using SEM analysis. We provide a cross-section of these studies in the domain of physical activity. Rovniak et al.^24^ presented the social cognitive model of the physical activity of college students from Virginia Polytechnic Institute and State University in the United States (USA). In their study, they measured self-efficacy, self-regulation, social support and outcome expectation at baseline and used them to predict physical activity 8 weeks later. They found that self-efficacy was the strongest determinants of physical activity, followed by self-regulation and social support. Similarly, Oyibo and colleagues^30,31^ modeled the physical activity of two different college student populations in Canada and Nigeria using the SCT as a theoretical framework. The authors measured all of the four main determinants of physical activity and used it to predict participants’ reported level of physical activity in the past 7 days. They found that self-efficacy and self-regulation had the strongest total effect on physical activity among the Canadian group, while social support and body image had the strongest total effect on physical activity among the Nigerian group.

Resnick^32^ presented a social cognitive model of the current exercise of older adults, living in a continuing care retirement community in the USA. The author found that self-efficacy, outcome expectation and prior exercise were among the strongest determinants of current exercise. Similarly, Anderson et al.^33^ modeled the physical activity of adults from 14 southwestern Virginia churches in the USA using the SCT as a theoretical framework. They found that self-regulation, self-efficacy and social support are the strongest determinants of the adults’ physical activity. Moreover, Anderson-Bill et al.^34^ investigated the determinants of physical activity among web-health users resident in the USA and Canada. Their model was based on the SCT and focused on walking as the target behavior. Specifically, they used pedometers to track participants’ daily steps and minutes walked over a 7-day period. They found that, overall, self-efficacy, self-regulation and social support were the determinants of participants’ physical activity, with self-efficacy being the strongest. Moreover, in the context of behavior modeling in a fitness app, Oyibo et al.^20^ investigated the perceived effect of behavior modeling on the SCT factors. They found that the perceived persuasiveness of the exercise behavior model design has a significant direct effect on self-regulation, outcome expectation and self-efficacy. More specifically, the effect was stronger on the first two SCT factors than on the third.

The major limitation of the above studies, apart from the last one reviewed, is that most of them used convenience samples, especially the student population. This may affect generalizing to a more diverse population sample.^24^ Moreover, none of the previous studies has investigated the SCT determinants of exercise behavior in the context of behavior modeling (in a fitness app), which is one of the main sources of self-efficacy^35^ including the other core social cognitive factors.^20^ Second, most previous studies did not use a mixed-method approach, comprising quantitative and qualitative analyses. Our study aims to fill this gap by using the mixed-method approach and providing evidence-based design guidelines for developing more effective fitness apps in the future.

## Methods

This section covers our research question/design, measurement instruments and the demographics of study participants.

### Research objective and design

The aim of our study is to investigate the determinants of bodyweight exercise behavior performance in the context of behavior modeling in fitness apps using the SCT as a theoretical framework. In particular, we aim to understand which of the SCT factors are the strongest drivers of exercise behavior performance and how the gender of the observers of the behavior moderates the various interrelationships among the SCT factors and the target behavior. More formally, our research questions can be stated as follows:

1. How are the SCT constructs interrelated in the context of behavior modeling in a fitness app?
2. Which of the SCT constructs are the strongest determinants of bodyweight exercise behavior performance?
3. Does gender moderate the interrelationships among the SCT constructs and exercise behavior performance?

To address the above research questions, we designed a hypothetical fitness app for encouraging exercise behavior on the home front. The app modeled two types of bodyweight exercise behaviors – push-ups and squats – that are commonly used in current fitness apps on the market. Apart from exercise type, the behavior models were designed taking race (black and white) and gender (male and female) into consideration. Figure 1 shows two of the eight versions of the behavior models, one of which was randomly administered to each participant who took part in the study. However, in this paper, we do not investigate the moderating effect of the design characteristics of the behavior models (i.e. race, gender and exercise type). In our survey, we requested participants to answer a number of SCT-related questions (see subsection Measurement instruments). Before answering the questions, the app was described to participants as follows:

Imagine you want to improve your personal health and fitness level. Given the challenges (e.g. time, cost, weather, etc.) associated with going to the gym regularly, the ‘Homex App’ has been created, say by health promoters in your neighborhood, to support your physical activity.

In particular, we intend to use the feedback from participants and the quantitative findings to inform our future PT intervention aimed at motivating people to exercise more (especially at home). Thus, in our survey, a snapshot of the mock-up of the proposed application (behavior models performing push-up and squat exercises shown in Figure 1) was presented to participants to elicit their feedback and investigate the interrelationships among the SCT factors and exercise behavior performance. Moreover, in this study, we assume that most of the respondents, with respect to susceptibility to persuasive technologies, are likely to be ‘January 1st’ people, who are open to change. Stibe and Larson^13^ described this group of people as ‘the most welcoming towards technology supported behavioral interventions’ designed to facilitate the achievement of the target behavior.

### Measurement instruments

We adapted our measurement instruments (see Table 1) from existing SCT scales in the literature. Exercise behavior was measured using the projected number of repetitions of bodyweight exercise (push-ups or squats) a participant could perform per week. Self-efficacy was measured using the scale proposed by Schwarzer and Renner.^36^ Social support, outcome expectation and self-regulation were adapted from Sallis,^37^ Wójcicki et al.^38^ and Rovniak et al.,^24^ respectively. Self-efficacy and social support used a Likert scale ranging from ‘not confident (0)’ to ‘confident (100)’, while outcome expectation and self-regulation ranged from ‘strongly disagree (1)’ to ‘strongly agree (5)’. In the SEM and descriptive statistics analysis, the 1–5 scale was rescaled to the 0–100% scale to ensure uniformity.^39^

**Table 1.** Measurement instruments.

Construct	Overall question and construct items
Exercise behavior	Assume you were to perform this exercise at home throughout the week.1. What is the average number of [name of exercise] do you think you can do per day?2. How many days per week do you think you can do the [name of exercise]?
Self-efficacy	How confident are you that you can complete at home the proposed weekly number of push-ups (entered previously) for the next 3 months. Even when you have worries and problems? Even if you feel depressed? Even when you feel tense? Even when you are tired? Even when you are busy?
Social support	How confident are you that you can complete at home the proposed weekly number of push-ups (entered previously) for the next 3 MONTHS, if a friend or family… Exercised with you? Offered to exercise with you? Gave you helpful reminders to exercise? Helped plan activities around your exercise schedule?
Outcome expectation	The [name of exercise] will… Improve my ability to perform daily activities. Improve my overall body functioning. Strengthen my bones. Increase my muscle strength. Improve the functioning of my cardiovascular system. Improve my social standing. Make me more at ease with people. Increase my acceptance by others.
Self-regulation	To achieve my proposed weekly average number of push-ups… I will set a goal. I will develop a series of steps to reach my weekly goal. I will keep track of my progress in meeting my goal. I will endeavor to achieve the set goal for myself. I will make goal public by telling others about it.

Outcome expectation comprises two lower-order constructs.Items 1 to 5 measure the physical outcome expectations, while items 6 to 8 measure the social outcome expectations.

### Participants

We submitted our research questionnaire to the authors’ university’s behavioral research ethics board. After its approval, we recruited participants from Amazon Mechanical Turk (AMT – a crowdsourcing platform based in the USA). We chose AMT because it is a platform through which researchers can gather data from diverse users with different demographic variables. Second, we chose AMT because it provides a mechanism that will allow for gathering research data that can be relied upon to a certain degree. For example, the platform allows researchers to reject responses they consider ‘poor’. This tends to reduce the chances of receiving invalid responses from questionnaire takers given the negative effect it has on their ‘overall reputation’ on the platform, which may prevent them from having the opportunity to participate in certain surveys in the future. This ‘quality assurance’ mechanism tends to increase the reliability of the gathered data on the platform compared to otherwise. In appreciation of participants’ time in taking the survey, which lasted for about 10–15 minutes, we compensated them with US$0.6 each. We paid a relatively conservative amount, lower than the average at the time (2017) because of our large sample size and to reduce the chances of the incentive affecting the overall responses due to certain takers answering the questionnaire solely for financial gains. A total number of 678 participants took part in the study. On cleaning, we were left with 659 participants for our SEM analysis. Table 2 shows the demographics of participants: 48.4% were women, while 51.6% were men – indicating that the gender distribution is almost balanced.

**Table 2.** Demographics of participants (*n*=659).

Criterion	Distribution (women, men) (319, 340)
Age (years)	18–24 (56, 70); 25–34 (134, 156); 35–44 (78, 76); 45–54 (37, 21); >54 (14, 17)
Education	Technical/trade school (44, 38); high school (64, 70); bachelor degree (152, 161); master’s degree (42, 54); doctorate degree (9, 6); other (8, 11)
Continent	North America (277, 269); Asia (19, 28); Europe (13, 19); South America (5, 10); Africa (3, 7); other (2, 7)
Country	Canada (105, 115); United States (188, 183); other (26, 42)
Race	White (253, 255); Black (22, 28); Brown (23, 33); other (21, 24)

### Research model

**Figure 2.**
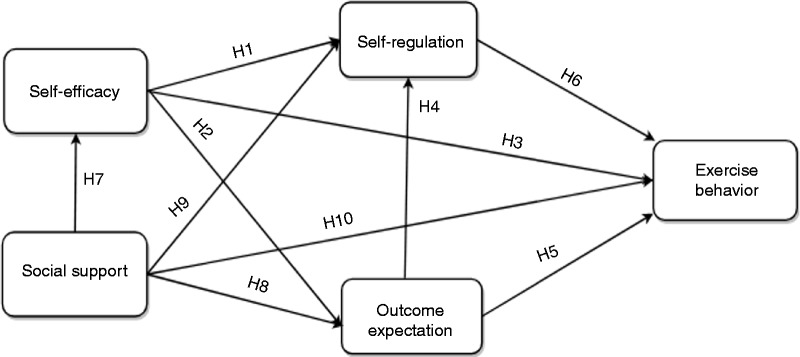
Hypothesized social cognitive model of exercise behavior.

Based on the existing empirical findings in the literature and, more specifically, the theoretical social cognitive model for health promotion proposed by Bandura,^40^ we formulated 10 hypotheses, as shown in Figure 2 – a follow-up SCT-based model to that of Oyibo et al.^20^ In the prior model, Oyibo et al. [20] found that the perceived persuasiveness of exercise behavior models significantly influences all of the three cognitive (internal) factors of the SCT: outcome expectation, self-regulation and self-efficacy. Apart from these three internal factors, we have added an external factor (social support) to our model shown in Figure 2 with the aim of uncovering how all four SCT factors impact exercise behavior in the context of behavior modeling.

The first and second hypotheses (H1 and H2) were based on the findings of Rovniak et al.^24^ in a study among 277 students of Virginia Polytechnic Institute and State University. In their study, which modeled students’ physical activity, they found that self-efficacy strongly influenced self-regulation and outcome expectation. Thus, in our contextualized study based on exercise behavior modeling, we hypothesize that the perceived self-efficacy of the observers of the behavior will positively influence their perceived self-regulation as well as their outcome expectations. The third hypothesis (H3) was informed by the self-efficacy theory of Bandura,^35,40^ which states that self-efficacy is the strongest (proximal) determinant of behavior in general. This theory was empirically validated by Oyibo.^30^ Among university student participants resident in Canada, Oyibo^30^ found that self-efficacy significantly influences their physical activity level. Based on this finding and the theory of self-efficacy, we hypothesize that, in the context of exercise behavior modeling, self-efficacy will directly influence users’ exercise behavior performance.

Furthermore, the fourth and fifth hypotheses (H4 and H5) are predicated on the social cognitive model proposed by Bandura,^40^ in which the author theorized that outcome expectations positively influence goals-based self-regulation and the target behavior in health promotion. These theorized relationships were validated by the empirical study carried out by Anderson-Bill et al.^34^ to model the physical activity behavior of web-health users. As a result, in our study, we hypothesize that outcome expectation will positively influence self-regulation and bodyweight exercise behavior performance. Similarly, the sixth hypothesis (H6) was informed by the theoretical social cognitive model of Bandura^40^ and the empirical validation of Anderson-Bill et al.^34^

Finally, the seventh and eighth hypotheses (H7 and H8) were informed by the findings of Anderson-Bill et al.^34^ in the eating domain, while the ninth and tenth hypotheses (H9 and H10) were informed by their findings in the physical activity domain. In the same study,^34^ the authors found that social support directly (positively) influenced self-efficacy, outcome expectation, self-regulation and physical activity (the target behavior). Hence, in our contextualized study, we hypothesize with respect to H7, H8, H9 and H10 that social support will positively influence all four social cognitive constructs as shown in Figure 2.

### Qualitative analysis

To uncover some of the main motivators and demotivators in users’ feedback with respect to the four hypothesized SCT determinants of exercise behavior (self-efficacy, self-regulation, outcome expectation and social support), we manually went through each comment to see what each participant was saying. In the discussion section, we provided a snippet of the relevant participants’ comments to support the validation of the respective hypotheses presented in Figure 2.

## Results

In this section, we present the descriptive statistics of our data, the evaluation of our measurement models, the analysis of our structural models and the multigroup analysis (MGA).

### Descriptive statistics

Table 3 shows the overall mean values for the SCT determinants and the target exercise behavior together with the standard deviations in brackets. Exercise behavior was operationalized as performance and calculated as a product of the number of repetitions of the target exercise (push-ups or squats) per day and the number of days per week. Thus, exercise behavior performance is measured in the number of reps/week. Overall, men projected more reps/week (267) than women did (142) with respect to the performance of the target behavior (push-ups/squats). Moreover, men rated their perceived self-efficacy and perceived social support higher than women did. In particular, with respect to self-efficacy, men (63.5%) had more confidence in their ability to perform bodyweight exercise than women (54.4%) did, leading to a higher exercise performance projection for men (267) than for women (142). Similarly, men (77.3%) had a stronger belief in social support from friends and family towards engaging in the target behavior than women (72.0%) did.

**Table 3.** Rating of social cognitive constructs.

Construct	Men	Women	Overall
Self-efficacy (%)	**63.5 (24.1)**	**54.4 (26.8)**	59.0 (25.8)
Social support (%)	**77.3 (21.3)**	**72.0 (26.0)**	74.7 (23.8)
Self-regulation (%)	70.7 (17.8)	72.9 (18.8)	71.8 (18.3)
Outcome expectation (%)	67.5 (16.0)	64.9 (15.3)	66.1 (15.7)
Exercise behavior (reps/week)	**267 (397)**	**142 (202)**	207 (324)

Bold values indicate men and women differ at *p* < 0.05.The SCT factors are on a scale from 0 to 100%.The values in brackets represent standard deviation.

### Measurement models

Our SEM analysis was carried out using the PLSPM package in R.^41^ We chose this software package because it is free and has well-documented resources on how to use it to build SEM models and analyze them in R Studio (e.g. Sanchez).^42^ Before carrying out the SEM analysis on the global, male and female structural models, we evaluated the respective measurement models based on the following required criteria: indicator reliability, internal consistency reliability, convergent validity and discriminant validity.^39,42,43^ We briefly discuss each criterion here.

#### Indicator reliability

All of the indicators in the measurement models had an outer loading greater than 0.7, except for ‘[name of exercise] will strengthen my bones’, which was less than 0.7. However, given the value was not less than 0.6, it was kept in the measurement models. Moreover, the outer loading for the indicator, ‘I will make my goal public by telling others about it’, was less than 0.5, so it was dropped from self-regulation in all three models.

#### Internal consistency reliability

We evaluated this metric for each construct using the composite reliability criterion, Dillon-Goldstein’s rho, which was greater than 0.7.

#### Convergent validity

We evaluated this criterion for each construct, using the average variance extracted, which was greater than 0.5.

#### Discriminant validity

We assessed this criterion using the cross-loading metric of each construct on the other constructs. Our results showed that there was no indicator which loaded higher on any other construct than the construct it was meant to measure.^39,42,43^

Finally, before building the respective models, we transformed the exercise behavior construct, which is based on the number of push-up/squat repetitions per week, to a normal distribution using the logarithm function (log_10_). We did this because the original distribution was highly skewed.

### Global model

**Figure 3.**
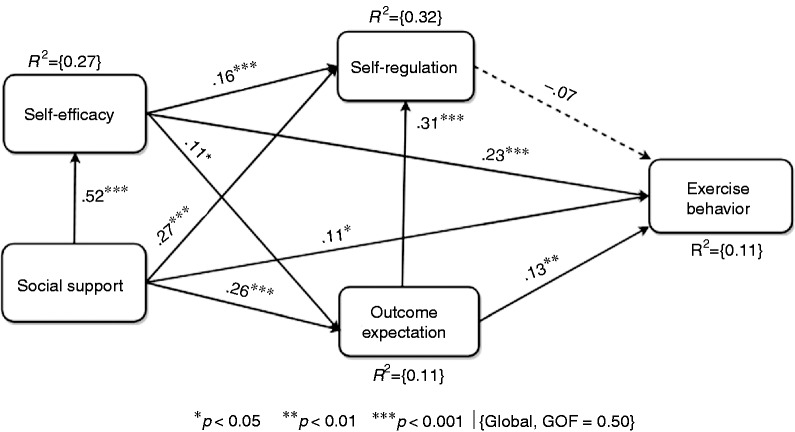
Global model of exercise behavior for the entire population sample.

Figure 3 shows the global model for the entire population sample together with the respective metrics that describe it: the goodness of fit (GOF) of the model, the coefficient of determination (R^2^) of the endogenous constructs and the path coefficient (β). The GOF represents how well the model fits its data, while R^2^ represents the amount of variance of an endogenous construct explained by the exogenous constructs. Finally, β represents the strength of the relationship between a pair of SCT constructs. In the global model, the GOF value is 50%, while the R^2^ value is 11%. This indicates that social support, self-efficacy and outcome expectation combined are able to explain 11% of the variance in exercise behavior. The low explanation of the target construct by the driver constructs is an indication that the global population sample is heterogeneous and/or there are other factors, which may account for the variance of exercise behavior, that are not captured in the model.

With respect to the interrelationships among the SCT constructs, the global model shows that self-efficacy (β = 0.23, *p* < 0.001), social support (β = 0.11, *p* < 0.05) and outcome expectation (β = 0.23, *p* < 0.01) directly (positively) influence exercise behavior. In particular, self-efficacy has the strongest direct effect on exercise behavior, while self-regulation (β =−0.07, *p* = n.s.) has the weakest (no significant) effect on exercise behavior. Moreover, the strongest direct effect in the model is that between social support and self-efficacy (β = 0.52, *p* < 0.001), indicating how strongly social support influences self-efficacy.

### Subgroup models

**Figure 4.**
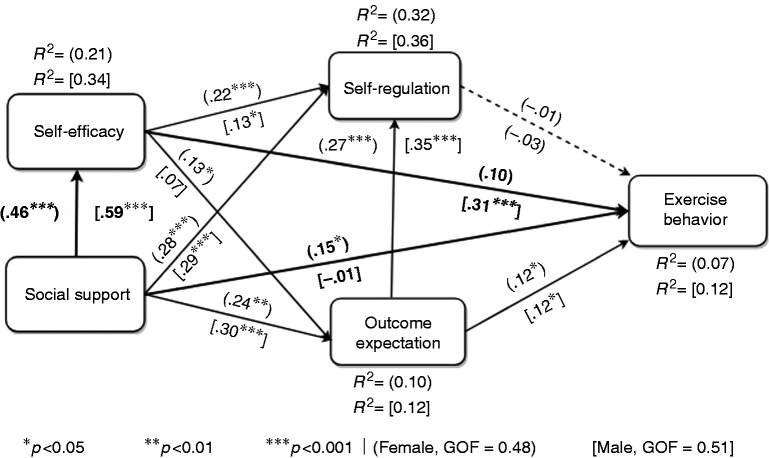
Subgroup models of exercise behavior for men and women (highlighted relationships indicate a significant gender difference (*p*<0.05) based on the multigroup analysis (MGA)).

We carried out a MGA to uncover the differences between the male and female groups, which make up the entire population sample. The results of our MGA indicated that there are significant differences between the male and female groups. Consequently, we built two different submodels for both genders, as shown in Figure 4. The circular brackets represent the female submodel, while the square brackets represent the male submodel. In particular, the differences between both submodels are with respect to the three interrelationships among three specific constructs: social support, self-efficacy and exercise behavior. On one hand, the MGA showed that men and women significantly differ with respect to the relationships between social support and self-efficacy, and between self-efficacy and exercise behavior, with these direct effects being stronger for the male group than the female group. On the other hand, the MGA showed that men and women significantly differ with respect to the relationship between social support and exercise behavior, with this direct effect being stronger for the female group than for the male group. Furthermore, we found that the variance of exercise behavior remains low still (12% for the male model and 7% for the female model). Again, this is an indication of an unobserved heterogeneity unexplained by gender difference and/or an indication of other uncaptured factors in the model. (In future work, we will attempt to uncover what the unobserved heterogeneity is, including other possible factors that may increase the explanation of the target behavior.)

### Total effect of SCT determinants on exercise behavior

**Figure 5.**
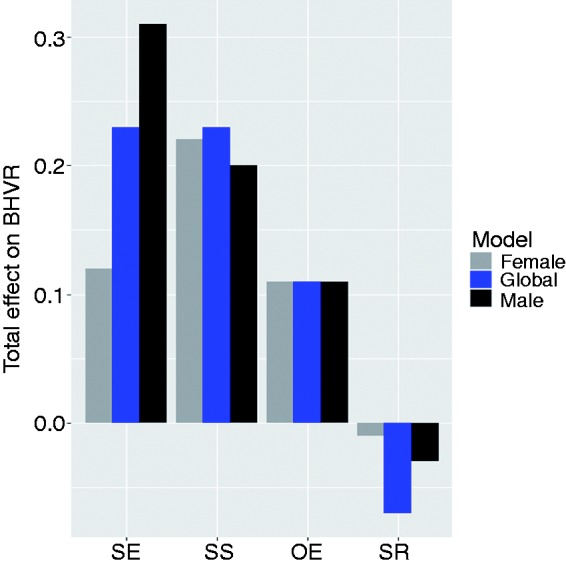
Total effects on exercise behavior (all of the total effects are significant at *p* < 0.05, except that of SR for all three models which are non-significant (*p* > 0.05) and that of OE for the male model which is marginally significant (*p* = 0.053). SE: self-efficacy; SS: social support; SR: self-regulation; OE: outcome expectation).

We carried out a total-effect analysis to determine which of the four determinants has the strongest overall influence and weakest overall influence on exercise behavior (the target behavior). The results of our analysis (Figure 5) showed that, at the global level, self-efficacy (β_T_ = 0.23, *p* < 0.001) and social support (β_T_ = 0.23, *p* < 0.001) have the strongest total effect on exercise behavior, followed by outcome expectation (β_T_ = 0.11, *p* < 0.05). However, as expected, self-regulation has no significant total effect on exercise behavior (β_T_ =−0.07, *p* < 0.05). At the subgroup level, for the male group, self-efficacy (β_T_ = 0.31, *p* < 0.001) has the strongest total effect on exercise behavior, followed by social support (β_T_ = 0.20, *p* < 0.001). In contrast, for the female group, social support (β_T_ = 0.22, *p* < 0.001) has the strongest total effect on exercise behavior, followed by social support (β_T_ = 0.12, *p* < 0.001). Furthermore, regardless of gender, outcome expectation has the third strongest total effect on exercise behavior, only that while it is completely significant for the female group (β_T_ = 0.11, *p* < 0.05), it is marginally significant for the male group (β_T_ = 0.11, *p* = 0.053). However, self-regulation has no significant total effect on exercise behavior for both the male and female groups, just as in the global model.

### Mediation analysis

We carried out a mediation analysis on the respective models to determine which of the relationships are mediated by a given construct. Our results showed that there are five mediated paths in the three SEM models (see Table 4). At the global level, self-efficacy (variation accounted for (VAF = 0.37) and outcome expectation (VAF = 0.22) partially mediate the influence of social support on exercise behavior. In the male submodel, self-efficacy (VAF = 0.54) partially mediates the influence of social support on exercise behavior. Similarly, outcome expectation (VAF = 0.21) partially mediates the influence of social support on self-regulation. Finally, in the female model, self-efficacy (VAF 0.26) partially mediates the influence of social support on self-regulation.

**Table 4.** Summary of the overall and gender-based findings.

Mediated path	Global	Men	Women
SS→SE→EB	**0.37**	**0.54**	–
SS→SE→SR	0.19	0.18	**0.26**
SS→SE→OE	0.15	–	0.17
SS→OE→EB	**0.22**	–	0.15
SS→OE→SR	0.19	**0.21**	0.16
SE→OE→EB	0.06	–	0.12
SE→OE→SR	0.15	–	0.12
SS→SR→EB	–	–	–
SE→SR→EB	–	–	–
OE→SR→EB	–	–	–

SS: social support; SE: self-efficacy; SR: self-regulation; OE: outcome expectation; EB: exercise behavior.‘–’ represents path in models that does not meet the condition to test for mediation using the variation accounted for by the indirect path metric.^38^ Moreover, bold values indicate partial mediation.

## Discussion

We have presented the results of a SEM analysis to answer our study’s research questions presented in the Research objective and design section. Table 5 summarizes all of our findings based on the verification of our 10 pre-stated hypotheses (H1–H10) and the gender-based MGA (F11–F13) based on an exploratory analysis. In particular, H1–H10 answer our first two research questions, while F11–F13 answer our third research question. At the global level, nine out of the 10 pre-stated hypotheses are validated, while, at the subgroup level, eight are validated. In the following subsections, we discuss the validation or non-validation of each of the 10 hypotheses and the three other findings based on the exploratory approach.

**Table 5.** Summary of the overall and gender-based findings.

SN	Group-specific (pre-stated) hypotheses	G	F	M
H1	The self-efficacy of the observers of behavior model positively influences their self-regulation.	✓	✓	✓
H2	The self-efficacy of the observers of behavior model positively influences their outcome expectation.	✓	✓	×
H3	The self-efficacy of the observers of behavior model positively influences their exercise behavior performance.	✓	×	✓
H4	The outcome expectation of the observers of behavior model positively influences their self-regulation.	✓	✓	✓
H5	The outcome expectation of the observers of behavior model positively influences their exercise behavior performance.	✓	✓	✓
H6	The self-regulation of the observers of behavior model positively influences their exercise behavior performance.	×	×	×
H7	The social support for the observers of behavior model positively influences their self-efficacy.	✓	✓	✓
H8	The social support for the observers of behavior model positively influences their outcome expectation.	✓	✓	✓
H9	The social support for the observers of behavior model positively influences their self-regulation.	✓	✓	✓
H10	The social support for the observers of behavior model positively influences their exercise behavior performance.	✓	✓	✓
	**Comparative (exploratory) findings**	**True**		
F11	The influence of social support on self-efficacy is stronger for men than women.	Yes		
F12	The influence of self-efficacy on exercise behavior performance is stronger for men than women.	Yes		
F13	The influence of social support on exercise behavior performance is stronger for women than men.	Yes		

G: global model; F: female model; M: male model.✓: supported/validated; ×: not supported/validated.

### Global validation of self-efficacy related hypotheses: H1, H2 and H3

Table 5 shows that the three hypotheses (H1, H2 and H3) related to self-efficacy are validated. This indicates that, in general, the higher the perceived self-efficacy of the observers of a modeled behavior, the higher their perceived outcome expectations and their belief in their self-regulation and the behavior performance. Moreover, based on our total-effect analysis, we found that self-efficacy (alongside social support) is the strongest determinant of bodyweight exercise behavior. This is consistent with the SCT, which holds that self-efficacy is the strongest (proximal) determinant of behavior change. According to the agentic perspective of positive psychology of Bandura,^44^ humans, as agents, have the power to effect changes in their environment by their action if and only if they believe in their capability to do so. At the core of the mechanism of agency is the belief in one’s capacity (i.e. self-efficacy) to influence the circumstances and the events of one’s own life, which, according to Bandura,^44^ is the ‘foundation of human motivation, well-being, and accomplishments‘ (p. 1). More specifically, Bandura argued that the self-efficacy beliefs of humans regulate their functioning through cognitive, motivational, emotional and decisional processes. He asserted that these self-efficacy beliefs, in general, influence the mindset of people (whether they will be optimistic in their thinking or pessimistic) and their outcome expectations (whether their efforts will yield favorable outcomes or not). Similarly, in our contextualized study of the SCT model of bodyweight exercise behavior performance, we found that the higher the self-efficacy of the observers of the behavior is, the higher becomes their outcome expectations (H2) and the belief in their ability to regulate themselves (H1) and perform the target behavior (push-ups/squats) ultimately (H3).

To support these quantitative findings with qualitative evidence, we provide a cross-section of the self-efficacy-related comments from participants, whose ratings of their outcome expectations, perceived self-regulation and exercise behavior performance in the survey, for the most part, reflect the level of their self-efficacy beliefs (see Table 6). For example, we see that P575 had a relatively high level of self-efficacy, as reflected in the qualitative comment, ‘I can do anything I set my mind to’, and the quantitative self-efficacy score of 100%. This high level of self-efficacy, in turn, might have influenced not only the participant’s outcome expectation (88%) and self-regulation (100%) but the projected exercise behavior performance (270 reps/week) as well. Compared to the overall average scores for women (see Table 3), we see that this participant’s scores in all four constructs are much higher. For example, for self-efficacy, outcome expectation, self-regulation and exercise behavior performance, the participant in question (P575) scored 100%, 88%, 100% and 270 reps/week, respectively, compared with the respective average scores of 54.4%, 64.9%, 72.9% and 142 reps/week for women.

**Table 6.** Self-efficacy as a motivator of exercise behavior performance.

ID	Exercise type	Comment	Profile
P575	Push-ups	I can do anything I set my mind to.	[Woman, SE = 100%, OE = 88% SR = 100% EB= 270 reps/week]
P590	Push-ups	I’m kind of lazy, so I’m not sure if I’ll actually do it. So, when I’m busy or stressed, the likelihood I’ll do it greatly decreases.	[Woman, SE = 16%, OE = 56% SR = 55% EB = 105 reps/week]
P100	Squats	Fitness is something I will actually make time for regardless how busy I am and it helps deal with stress and depression so it’s something I already do on a daily basis.	[Man, SE = 100%, OE = 88% SR = 80% EB = 300 reps/week]
P144	Squats	There is nothing to motivate me to actually do the workout its boring and if I have better things to do I won’t do it.	[Man, SE = 16%, OE = 16% SR = 55% EB = 50 reps/week]

SE: self-efficacy; SR: self-regulation; OE: outcome expectation; EB: exercise behavior.

In contrast, for P590, who believed ‘I am kind of lazy, so I’m not sure if I’ll actually do it…’, as reflected in the low self-efficacy score (16%), compared with the respective average scores for women, her outcome expectations (56% vs. 64.9%), belief in her self-regulation (55% vs. 74.9%) and exercise behavior performance (105 reps/week vs. 142 reps/week) were correspondingly impacted (negatively) as well. In sum, the two qualitative comment-based examples (P575 and P100) concerning push-up exercises, and the other two examples (P590 and P144) concerning squat exercises (shown in Table 6) confirm the validation of H1, H2 and H3 in the global model (shown in Figure 3). In other words, they confirm that the higher (or lower) the perceived self-efficacy of the observers of a modeled behavior (push-ups or squats), the higher (or lower) their outcome expectations, their belief in their ability to regulate themselves and perform the targeted exercise behavior. Moreover, in the context of personal susceptibility to persuasive technologies, as theorized by Stibe and Larson,^13^ P575 and P100 can be described as potential (‘January 1st’) users of the proposed PT intervention (fitness app), who are open and willing to change, while P590 and P144 as non-potential (‘self-contained’) users, who may not want or be willing to change.

### Global validation of outcome-expectation related hypotheses: H4 and H5

The global model (Figure 3) shows that the hypotheses (H4 and H5) related to outcome expectation are supported by the quantitative data as summarized in Table 5. In other words, the higher the outcome expectations of the observers of the modeled behavior, the more likely they are to regulate themselves (H4) or perform the exercise behavior (H5). The verifications of these hypotheses are evident in the qualitative data (outcome expectation-related comments provided by participants) as well. Table 7 shows the comments of four participants (two with high outcome expectations and two with low outcome expectations) together with their SCT belief profile on self-regulation and exercise behavior performance.

**Table 7.** Outcome expectation as a motivator of exercise behavior.

ID	Exercise type	Comment	Profile
P496	Push-ups	Push-ups will be good for me, make me stronger and make it easier for me to do regular things like lifting boxes and such.	[Woman, OE = 78%, SR = 95%, EB = 700 reps/week]
P654	Push-ups	I really can’t see it doing much other than making your arms stronger. Maybe you’d lose a tiny bit of weight. Nothing more, though.	[Man, OE = 56%, SR = 40%, EB = 120 reps/week]
P187	Squats	Physical exercises make me feel better about my look, my look gives me self-confidence.	[Woman, OE = 66%, SR = 75%, EB = 910 reps/week]
P212	Squats	This exercise may slightly help but I don’t think it would be anything life changing.	[Woman, OE = 28%, SR = 25%, EB = 20 reps/week]

SE: self-efficacy; SR: self-regulation; OE: outcome expectation; EB: exercise behavior.

P496, for example, stated ‘push-ups will be good for me, make me stronger and make it easier for me to do regular things like lifting boxes and such’. This positive belief in the physical benefit of push-up exercise, expressed in words, is reflected in the participant’s outcome expectation belief score (78%), self-regulation belief score (95%) and projected exercise behavior performance (700 reps/week). Similarly, P187 believed ‘physical exercises make me feel better about my look, my look gives me self-confidence’. This positive belief in the social benefit of exercise, expressed in words, is reflected in the participant’s outcome expectation belief score (66%), self-regulation belief score (75%) and exercise behavior performance (910 reps/week). In contrast, P654 and P212 did not have outcome expectations as positive as those of P496 and P187. This is evident in their respective comments as well as their outcome expectation belief scores shown in Table 7. For example, while P654 believed ‘I really can’t see it doing much other than making your arms stronger. Maybe you’d lose a tiny bit of weight. Nothing more, though’, P212 believed the squat exercise ‘may slightly help but I don’t think it would be anything life changing’. These negative outcome expectations, as reflected in the outcome expectation belief score (56% and 28%) for P654 and P212, respectively, went as far as affecting their belief in their self-regulation (40% and 25%) and performance of the target exercise behavior (120 reps/week and 20 reps/week). (These scores in brackets (and the subsequent similar ones) correspond to the respective participants aforementioned.) These scores in all three constructs (OE, SR and EB) for both participants are relatively lower than the respective overall average scores shown in Table 3: outcome expectation (66.1%), self-regulation (71.8%) and exercise behavior (207 reps/week). Based on these results, coupled with the qualitative evidence, we conclude that our fourth and fifth hypotheses (H4 and H5), shown in Table 5, are supported by the data. Thus, the higher (or lower) the outcome expectations of the observers of a modeled behavior (push-up or squat), the higher (or lower) their belief in their ability to regulate themselves or perform the targeted exercise behavior.

### Global validation of self-regulation related hypotheses: H6

The global model (Figure 3) shows that the hypothesis (H6) related to self-regulation is not supported by the quantitative data. In other words, the higher the observers’ belief in their ability to regulate themselves (i.e. set goals) may not necessarily lead to a higher exercise behavior performance (i.e. the number of repetitions of push-ups/squats per week). This finding, in light of other findings (see Figure 5), suggests that setting goals alone may not be enough to bring about the eventual performance of the target exercise behavior; other factors, such as self-efficacy, outcome expectations, social support, etc., may be necessary. One possible explanation for why self-regulation belief does not influence exercise behavior performance is that, in a real-life setting, people may end up not meeting their set goals given a number of reasons, which include lack of time, lack of motivation, lack of self-efficacy, lack of social support, etc. This is evident in the following comments from two of the study’s participants:

I usually have a hard time meeting my workout goals even if I set small goals. My busy life gets in the way and I just forget about doing it. (P337, push-ups)

If I have someone to help me stay on track, I might be more motivated to stick to my goals. (P387, push-ups)

P337, for example, alluded to lack of time (busy schedule) as the reason for not meeting set goals, even small goals. On the other hand, P387 alluded to lack of social support as the reason for not sticking to his/her goals. These comments reveal that setting of goals alone may not be enough motivation for someone to engage in a given exercise behavior; there have to be other drivers such as personal motivation (self-efficacy) – irrespective of busy schedules or other life challenges – and social support.^11^

### Global validation of social support related hypotheses: H7, H8, H9 and H10

In our global model (Figure 3), we showed that both the direct relationships social support has with self-efficacy, self-regulation, outcome expectation and exercise behavior are significant. Hence, our last four hypotheses (H7, H8, H9 and H10) are supported by the global data-driven model. These hypotheses state that the higher the observers’ belief in *s*ocial support, the higher will be their self-efficacy, outcome expectation, self-regulation beliefs and projected exercise behavior performance. Moreover, these hypotheses are supported quantitatively (in the SEM model) and qualitatively (see participants’ response and profile shown in Table 8). In particular, we see that P595 and P10 believed in social support and its power to influence their exercise behavior performance, while P430 and P53 did not. These opposing beliefs in the positive benefit of social support in the performance of the target behavior went as far as influencing (directly) not only the three other determinants of exercise behavior but the target behavior as well.

**Table 8.** Social support as a motivator of exercise behavior.

ID	Exercise type	Comment	Profile
P595	Push-ups	If I have someone keeping me accountable I would be more inclined to perform.	[Man, SS = 100% SE = 66%, OE = 100% SR = 100% EB = 700 reps/week]
P430	Push-ups	I do not like working out with friends and family. If they pushed me I would feel pressure and most likely abandon the whole idea.	[Woman, SS = 28% SE = 32%, OE = 56% SR = 60% EB = 75 reps/week]
P10	Squats	Having the support of family/friends will only help me feel more motivated to exercise.	[Woman, SS = 100% SE = 76%, OE = 84% SR = 85% EB = 500 reps/week]
P53	Squats	I exercise alone. Someone reminding me or telling me to pisses me off and wouldn’t encourage me at all.	[Woman, SS = 0% SE = 66%, OE = 41% SR = 35% EB = 150 reps/week]

SS: social support; SE: self-efficacy; SR: self-regulation; OE: outcome expectation; EB: exercise behavior.

On one hand, P595 and P10 believed in the power of social influence as evident in their respective comments: ‘If I have someone keeping me accountable I would be more inclined to perform’ and ‘having the support of family/friends will only help me feel more motivated to exercise’. Consequently, their respective beliefs in social support (100% and 100%) influenced their self-efficacy belief (66% and 76%), outcome expectations (100% and 84%), self-regulation belief (100% and 85%) and projected exercise behavior performance (700 reps/week and 500 reps/week). These findings – which represent an overall positive effect of perceived social support on exercise behavior in our study – replicate prior findings in a self-report study among a Pakistani population. Samir et al.^11^ found that lack of spouse and family support constitutes one of the main barriers to physical activity. In particular, the authors found that people from extended families are more likely to be inactive than people from nuclear active families.

On the other hand, P430 and P53 did not believe in the efficacy of social influence. This is evident in their respective comments: ‘I do not like working out with friends and family. If they pushed me I would feel pressure and most likely abandon the whole idea’, and ‘I exercise alone. Someone reminding me or telling me to pisses me off and wouldn’t encourage me at all’. Consequently, compared with the overall average scores of the respective constructs shown in Table 3, their relatively low belief in social support (28% and 0%) influenced (decreased) their respective self-efficacy belief (32% and 66% – an exception), outcome expectation (56% and 41%), self-regulation belief (60% and 35%) and projected exercise behavior performance (75 reps/week and 150 reps/week). Based on this quantitative evidence, coupled with that in the global model (shown in Figure 3) and qualitative evidence (presented in Table 8), we conclude that H7, H8, H9 and H10, as shown in Table 5, are supported.

### Gender differences: F11, F12 and F13

Our MGA (Figure 4) shows that men and women significantly differ with respect to H3, H7 and H10. These differences are summarized as F12, F11 and F13, respectively, in Table 5. First, the influence of social support on self-efficacy is stronger for men than for women F11. This suggests that the social support received by men is more likely to influence their self-efficacy in comparison with that received by women. This is also evident in mens’ stronger belief in social support (77.3%) in their performance of bodyweight exercise compared to that of women (72.0%) as shown in Table 3. Second, the influence of self-efficacy on the exercise behavior performance is stronger for men than for women F12. In fact, while this relationship is significant for the male group, it is not for the female group. In other words, mens’ belief in their self-efficacy influenced their projected performance of the target exercise behavior, but this is not the case for women. This finding may not be surprising, given that, generally, men have more confidence in their ability to perform physical activity, as evident in Table 3, in which the overall average self-efficacy of men (63.5%) is significantly higher than that of women (54.4%). Finally, the influence of social support on exercise behavior performance is stronger for women than for men (F13). In fact, while this relationship is significant for women it is non-significant for men (see Figure 3). This suggests that, in practice, the social support women receive can directly influence their performance of the target exercise behavior, unlike men, for whom self-efficacy partially mediates the relationship between both constructs as shown in Table 4.

### General guidelines for PT design based on main findings

Based on the validated hypotheses and the qualitative comments provided by participants, we provide a number of general guidelines to inform the PT design of health interventions to encourage user engagement in bodyweight exercise behavior to improve health and wellbeing. In particular, the guidelines are based mainly on the significant determinants of bodyweight exercise behavior, which include self-efficacy, social-support and outcome-expectation. More specifically, these strategies are targeted at enhancing self-efficacy because it is the strongest proximal determinant of behavior change as evident in the SEM model shown in Figure 3.

#### Guideline 1. Increase the enactive mastery experience of users (self-efficacy)

Make users be aware of their previous accomplishment of the target exercise behavior, for example, the achievement of a previous goal. Bandura^35^ regarded the enactive mastery experience as the strongest source of self-efficacy. According to the self-efficacy theory,^35^ each success attained by the user builds more confidence in his/her ability to repeat the achievement of the initial success. This awareness of previous success, in the face of difficulties or challenges, can serve as a booster of the user’s confidence in accomplishing the target behavior once again. The following snippets of comments from participants are examples of users being so confident in themselves due to their past performance and achievements:

I currently work out at least 3 days a week at a gym so I’m extremely confident (from experience) that I can perform the above workout. (P3, squats)

I am completely confident I can because I already do squats every day. (P5, squats)

Doing 100 push-ups has never been a problem for me. I would just need to schedule it into every day. (P617, push-ups)

#### Guideline 2. Allow users to collaborate with and motivate one another to perform the target behavior (social support)

Allow users to collaborate with and support one another to increase their motivation to perform the target exercise behavior. Persuasive strategies, such as peer-based or group-based cooperation, can be used as an effective social influence strategy to encourage users to perform the target behavior. Peer/social support (e.g. cooperation) is more likely to have a stronger direct effect on womens’ exercise behavior than that of men (see Figure 4). Competition could be used as well for certain users who are motivated by competition, for example, men and/or younger people.^45,46^ In particular, with respect to cooperation, users feel a sense of accountability^47^ and, as a result, tend to avoid disappointing their collaborative partners once they have committed themselves. The following are examples of participants’ comments that attest to the feeling of accountability in particular and the efficacy of cooperation and competition strategies in general:

Having someone relying on me to do an activity with them is the number one way for me to commit to actually doing it. Not letting someone down is a very strong motivator. (P371, push-ups)

I think the support system would make a big difference in motivation. It gives accountability, as well as competition. (P9, squats)

Working with a friend motivates you to complete the task and has an added level of competition. I am less likely to skip my workout if I have a friend present who is also doing it. (P51, squats)

I am easily motivated by others rather than by myself. If my family, friends or coworkers did the exercises with me, I would feel the pressure to do them as well. I am never one to turn down a challenge, so creating a game out of it or having a competition with it. (P68, squats)

It’s always easier when you have a friend around to encourage you, push you and share your pain. Competition is also encouraging. (P 556, push-ups)

#### Guideline 3. Model the behavior so that users can observe its performance and visualize its outcomes (outcome-expectation)

Provide users with a means to observe the performance of the behavior and its outcomes. One way to achieve this is the use of behavior modeling (see Figure 1) or simulation strategy, which models the causes and effects of the performance of the behavior. Behavior modeling is regarded as the second strongest source of self-efficacy.^48^ According to Bandura,^35^ when the user observes a peer or role model successfully perform the target behavior, he/she will feel more confident in him/herself to perform it as well. The following comments from participants on the visual design of the behavior models, shown in Figure 1, attest to the potential effectiveness of behavior modeling as a persuasive strategy for motivating the performance of a target exercise behavior:

Exercise in the mentioned video looks like it makes a person stronger and fit. (P284, man, push-ups)

Having this app do exercise with you makes me feel more inclined to exercise. (P657, push-ups)

Watching this gives me the impression to do this. (P574, push-ups)

## Summary and contributions

In this section, we provide the summary of our main findings, which double as our contributions to the existing body of knowledge. In particular, we contribute to knowledge in the field of theory-informed PT health interventions. Our main findings and contributions can be summarized as follows:

- We validated the SCT model in the context of behavior modeling of bodyweight exercise behavior in a fitness app prototype aimed at motivating behavior change.
- We showed that self-efficacy and social support, followed by outcome expectation, are the strongest determinants of bodyweight exercise behavior.
- We showed that self-regulation (i.e. goal-setting), when self-efficacy, social support and outcome expectation are controlled for, does not have a significant influence on bodyweight exercise behavior.
- We showed that the direct effect of social support on bodyweight exercise behavior is significantly stronger for women than for men.
- We showed that the direct effect of social support on self-efficacy and the direct effect of self-efficacy on bodyweight exercise behavior are significantly stronger for men than for women.
- We provided a set of general guidelines, based on the significant SCT determinants of bodyweight exercise behavior, for the design of persuasive apps aimed at motivating behavior change in the fitness domain.

### Limitations and future work

Our study has a number of limitations. The first and foremost limitation of the study is that it is based on a hypothetical fitness app – and not an actual fitness app modeling exercise behaviors. Second, our findings are based on the impact of perceived belief (SCT) constructs (such as self-efficacy) on the performance of exercise behavior. For these reasons, our findings, which are based on self-report, may not generalize to a real-life application setting in which the study participants would have to answer questions on the SCT factors and subsequently use the health app over a period of time, with their exercise performance and activities being tracked. For example, perceived self-regulation, which has a non-significant impact on participants’ projected exercise performance, may turn out to have a significant impact on the latter in a real-life fitness application. Thus, to bridge this gap in our current study, we recommend that the replication of our validated SEM model be verified using data gathered from a real-life health application. The third limitation of our study is that it focused specifically on participants resident in North America, who were mostly Canadian and American citizens. This may threaten the generalizability of our findings to users from other continents, countries and cultures. Thus, in future research efforts in the area of PTs for promoting exercise behavior, we recommend that our study be conducted among other demographics to uncover the generalizability of our findings.

## Conclusion

Behavior modeling is one of the main behavior change techniques through which humans observe the actions and consequences of the behaviors of other people, and ultimately acquire the necessary knowledge and skills to engage in the modeled behavior. In this paper, using SCT as a theoretical framework of behavior change, we investigated, in the context of behavior modeling, which of the SCT (belief) constructs are the strongest determinants of bodyweight exercise behavior performance on the home front. To uncover the determinants of exercise behavior performance, we carried out an empirical study among 659 participants resident in the USA and Canada, using behavior models demonstrating push-up and squat exercises as a case study. The results of our SEM analysis showed that perceived self-efficacy and perceived social support are the strongest determinants of bodyweight exercise behavior, followed by outcome expectation. Moreover, in our SEM model, perceived self-regulation turns out to have a non-significant influence on bodyweight exercise behavior. Moreover, our results showed that the direct and total effect of self-efficacy on bodyweight exercise behavior is stronger for men than for women, while the direct and total effect of social support on bodyweight exercise behavior is stronger for women than for men. Finally, based on the significant SCT determinants, we recommend a set of design guidelines to inform the implementation of persuasive health apps to drive behavior change in the fitness domain.
